# Association between Inflammatory Factors in the Aqueous Humor and Hyperreflective Foci in Patients with Intractable Macular Edema Treated with Antivascular Endothelial Growth Factor

**DOI:** 10.1155/2021/5552824

**Published:** 2021-06-07

**Authors:** Min Li, Jing Li, Kaichuan Chen, Jia Wang, Minjie Sheng, Bing Li

**Affiliations:** ^1^Department of Ophthalmology, Zhongshan Hospital Affiliated with Fudan University, Shanghai, China; ^2^Department of Ophthalmology, Yangpu Hospital, Tongji University School of Medicine, Shanghai, China

## Abstract

**Background:**

To evaluate the correlations between the inflammatory factors in the aqueous humor and hyperreflective foci (HRF) in patients with intractable macular edema treated with antivascular endothelial growth factor (anti-VEGF).

**Methods:**

This study included 17 patients with intractable macular edema (ME) treated with anti-VEGF agents. Inflammatory factors in the aqueous humor were measured by the Cytometric Beads Array before injection, and the numbers of HRF pre- and post-anti-VEGF treatment were counted from four different directions (90 degrees, 45 degrees, 180 degrees, and 135 degrees) in the SD-OCT images, respectively, before treatment and one month after treatment. The correlations between inflammatory factors and the numbers of HRF were assessed.

**Results:**

The numbers of HRF were reduced significantly after anti-VEGF treatment. The change in the HRFs at the 90-degree location was significantly positively correlated with IL-8 and VCAM-1. The change of all HRFs was significantly positively correlated with IL-8. The HRFs before the treatment also had a positive correlation with IL-8 and VCAM-1.

**Conclusion:**

After anti-VEGF treatment, the numbers of HRF in intractable ME declined greatly. The higher the levels of IL-8 and VCAM-1 before treatment, the more significant the reduction of HRF after anti-VEGF treatment, which indicated that HRF could be an effective noninvasive imaging indicator for evaluating the effect of anti-VEGF on intractable macular edema. The OCT images at the 90-degree location could better show the inflammatory reaction of patients and also had better clinical significance for the prognosis evaluation of ME associated with inflammation.

## 1. Introduction

The macular is the most sensitive location of vision, and macular edema (ME) is one of the main reasons for visual impairment in the presence of macular lesions. In clinical circumstances, ME is mainly caused by diabetic retinopathy [1] and retinal vein occlusion [2]. Research has revealed that the major cause of ME is the production of vascular endothelial growth factor (VEGF) [3]. Anti-VEGF agents are currently the most cutting-edge treatment options for ME. However, some of the patients treated with the same anti-VEGF drug for ME have poor visual outcomes [3]. Moreover, after several treatments with anti-VEGF, ME is still existing or recurring, known as intractable ME [4]. For this reason, clinicians have to find other methods of predicting the final visual outcomes of intractable ME.

Hyperreflective foci (HRF) is defined as “discrete, well-circumscribed dots with equal or greater reflectivity than the RPE band” in the images of spectral domain optical coherence tomography (SD-OCT) [5, 6]. HRF can be found throughout all retinal layers, but with special accumulations in the intraretinal cystoid spaces. Moreover, HRF has been reported in various retinal diseases, such as retinal vein occlusion [7], diabetic macular edema (DME) [8], and neovascular age-related macular degeneration (AMD) [9]. Some researchers reported that HRF in the AMD and diabetic maculopathy patients showed dynamic changes after anti-VEGF therapy [10–12]. Recent studies demonstrated that HRF could predict the final visual outcome in ME patients with anti-VEGF agents [13, 14]. Therefore, HRFs can be a new biomarker in patients with ME treated with anti-VEGF agents. However, it was reported that in a group of intractable ME patients treated with anti-VEGF agents, the mechanism underlying the change in HRF remained unclear.

In this study, we compared the changes in numbers of HRF and measured its correlation with inflammatory factors in intractable ME patients treated with anti-VEGF drugs, in order to find the possible roles and mechanism of HRF in the clinical follow-up of intractable ME.

## 2. Methods

### 2.1. Subjects

This study included 17 patients with intractable ME, who had been treated with anti-VEGF agents at least twice based on a previous study [4]. The last intravitreal injection was at least two months before extraction of aqueous humor. Only ME related with diabetic retinopathy or retinal vein occlusion was taken into account and recorded in this study. All the eyes of the studied subjects with the following symptoms were excluded: ME with hard exudate at the macula, epiretinal membrane or foveal traction, vitreous hemorrhage or pre- and subretinal hemorrhage, uveitis, glaucoma, or other retinal diseases. These data were collected at the Department of Ophthalmology, Yangpu Hospital, Tongji University School of Medicine, Shanghai, between March 2019 and October 2019, with the approval of the Ethics Committee of the Hospital (ethical approval no. LL-2018-ZRKX-031). All the processes were performed in accordance with the tenets of the Declaration of Helsinki. All the subjects were informed about the purpose and study method and signed their informed consent before examination. Using a 23 gauge needle, we extracted 100 *μ*l aqueous humor from every intractable ME patient before an intravitreal injection of anti-VEGF.

The levels of inflammatory factors (VEGF, BFGF, IL-6, IL-10, IL-8, and vascular cell adhesion molecule-1 (VCAM-1)) in the aqueous humor were analyzed and quantified using Cytometric Beads Array (BD Biosciences, USA) and flow cytometry.

### 2.2. Characteristic Analysis of HRF in the SD-OCT Images

**Figure 1:**
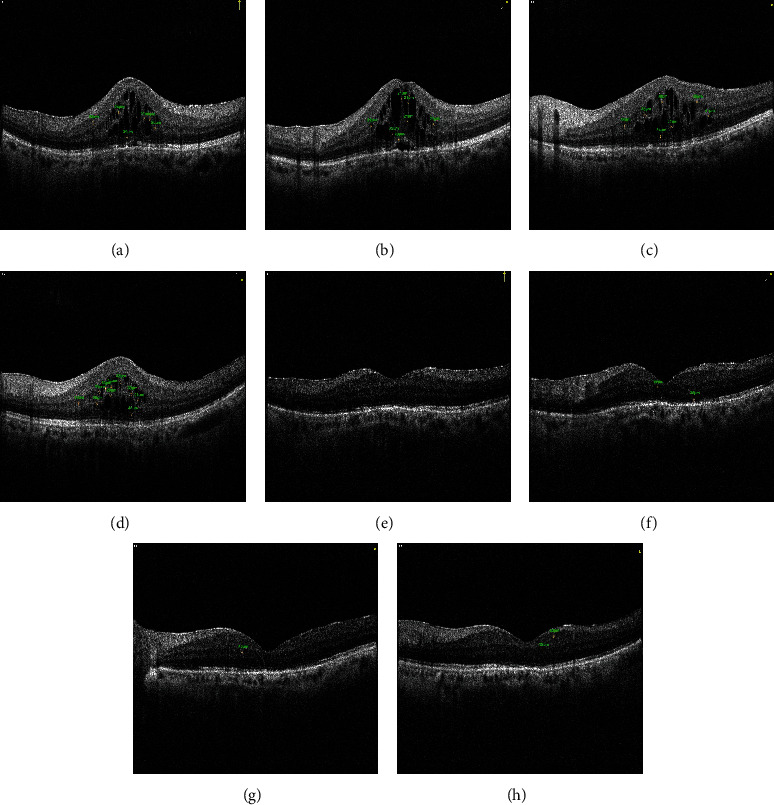
The change of HRFs in a female patient at OCT images at four locations before and after anti-VEGF treatment (90-degree, 45-degree, 180-degree, and 135-degree). Before this time of collecting aqueous humor and anti-VEGT treatment, this patient had already accepted three times of anti-VEGT treatment whose ME was still persistent. (a) Image at 90 degrees before treatment. (b) Image at 45 degrees before treatment. (c) Image at 180 degrees before treatment. (d) Image at 135 degrees before treatment. (e) Image at 90 degrees after treatment. (f) Image at 45 degrees after treatment. (g) Image at 180 degrees after treatment. (h) Image at 135 degrees after treatment.

A volume scan covering a 6 × 6 mm area of the macular area centered on the fovea was acquired using SD-OCT (RTvue XR, OPTOVUE, USA). HRF in the SD-OCT images is a small, well-circumscribed, dot-shaped lesion with similar or higher reflectivity than RPE in all retinal layers. The range of diameters of HRF was limited to 20 to 50 *μ*m [5, 6]. Therefore, we excluded small noise signals and large hyperreflective lesions considered to be hard exudates. We collected OCT images of each patient at four locations (90-degree, 45-degree, 180-degree, and 135-degree), respectively, before and one month after anti-VEGF treatment (Figure 1). The numbers of HRF were counted separately by two independent observers, and then, an average value was taken.

### 2.3. Statistical Analysis

The statistical analysis was performed with SPSS software version 19.0 (SPSS, Inc., Chicago, IL, USA). All values are reported as mean ± standard deviation (mean ± SD). A paired Student's *t*-test was used to compare the differences in HRF pre- and posttreatments with anti-VEGF. The correlations between the levels of inflammatory factors and the change in the numbers of HRF were assessed with Pearson's or Spearman's correlation analysis. A value of was considered statistically significant.

## 3. Results

17 eyes of 17 subjects were recorded and enrolled in this study (8 males and 9 females, mean ± SD, 58 years ± 12 years). All the subjects had been treated with anti-VEGF agents at least twice prior to their aqueous humor measurement. The anti-VEGF agent names, the previous injections of anti-VEGF, and the inflammatory factors in aqueous humor for this treatment are listed in Table 1. Foveal thickness and HRFs in OCT images before and after anti-VEGF treatment are shown in Table 2.

### 3.1. The Changes of Fovea Thickness and the Numbers of HRF after Anti-VEGF Treatment

**Figure 2:**
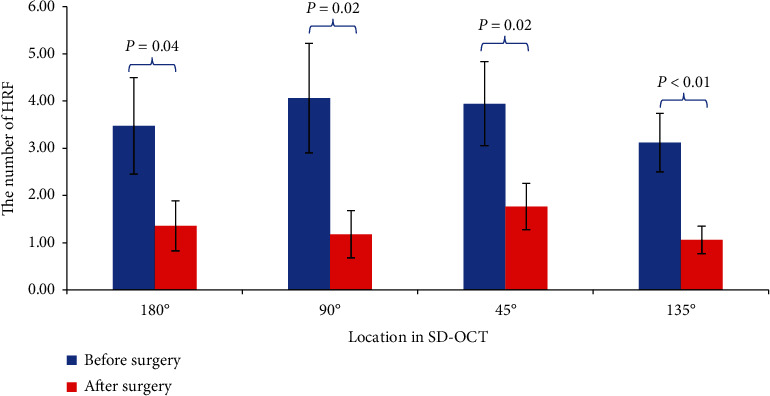
Comparison of the numbers of HRF at four locations in SD-OCT images before and after anti-VEGF treatment.

After treatment, the fovea was significantly thinner than before (*P* < 0.01), which indicated that after more than two times anti-VEGF treatments, macular edema had been effectively controlled. In the meantime, the numbers of HRF significantly decreased at four locations (90-degree, 45-degree, 180-degree, and 135-degree) after the treatments compared with before the treatment. (Figure 2).

### 3.2. Correlation between the Changes of HRF and the Inflammatory Factors in the Aqueous Humor

**Figure 3:**
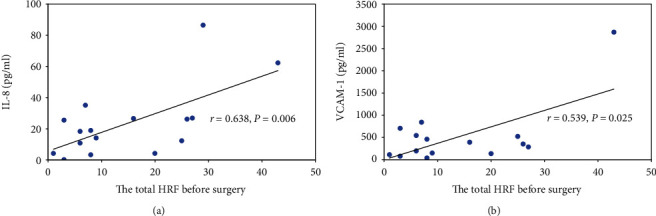
Correlation between the total HRF before anti-VEGF treatment and expression of inflammatory factors (IL-8 and VCAM-1) in the aqueous humor.

**Figure 4:**
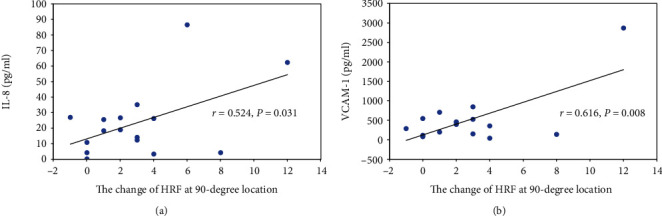
Correlation between the change of HRFs at 90-degree location after anti-VEGF treatment and expression of inflammatory factors (IL-8 and VCAM-1) in the aqueous humor.

In this study, the numbers of total HRF before the treatments had a positive correlation with IL-8 (*r* = 0.638, *P* = 0.006) (Figure 3(a)) and VCAM-1 (*r* = 0.539, *P* = 0.025) (Figure 3(b)), which indicated that the higher the levels of IL-8 and VCAM-1, the more HRF were found in their OCT images. Also, the changes of HRFs after treatments at the 90-degree location of the SD-OCT images were significantly positively correlated with the levels of IL-8 (*r* = 0.524, *P* = 0.031) (Figure 4(a)) and VCAM-1 (*r* = 0.616, *P* = 0.008) (Figure 4(b)). In contrast to the 90-degree location, the changes in numbers of HRF after treatments at the 45-degree, 135-degree, and 180-degree locations in the OCT images showed no correlation with IL8 or VCAM-1 (*P* > 0.05).

**Figure 5:**
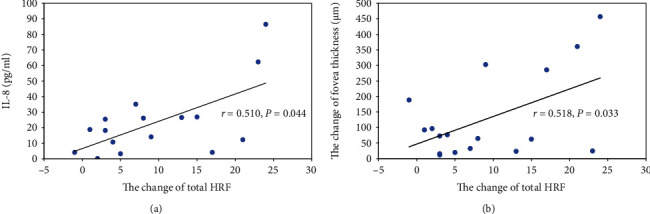
Correlation between the change of total HRFs after anti-VEGF treatment, levels of IL-8 in the aqueous humor and the change of fovea thickness.

More interestingly, we found that the changes of total HRFs after treatments were also significantly positively correlated with its levels of IL-8 (*r* = 0.510, *P* = 0.044) (Figure 5(a)) and the changes of fovea thickness (*r* = 0.518, *P* = 0.033) (Figure 5(b)), which indicated that, if the levels of IL-8 and VCAM-1 in aqueous humor were higher before treatments, the numbers of HRF should decrease more after anti-VEGF treatments. In addition, there were no significant correlation between the changes of HRFs and other inflammatory factors (VEGF, BFGF, IL-6, and IL-10) (*P* > 0.05).

## 4. Discussion

ME is clinically defined as an accumulation of serous fluid within the neurosensory retina, with increased thickness of the central retina. Some researches have shown that the leakage from the choroidal new vessels, predominantly VEGF-induced, may produce a large accumulation of fluid under the neurosensory retina [15]. Anti-VEGF has now become the cutting-edge treatment choice for ME, due to its excellent visual and anatomic improvement [16]. However, along with the widespread application of this treatment scheme, clinicians have found that some patients with the same intractable ME did not achieve satisfactory visual recovery postoperatively. Some indicators are therefore needed to evaluate the outcome of postoperative treatments in advance in intractable ME patients. Prior researches found that HRF showed dynamic changes after anti-VEGF therapy in patients with neovascular DME [16], AMD [17], and retinal vein occlusion [18]. In our study, HRFs in the SD-OCT were found scattered throughout all retinal layers, primarily around fluid accumulation in the intraretinal cystoid spaces. This study found that patients with intractable ME were taking a turn for the better and HRFs in four directions were significantly reduced after receiving more than two times anti-VEGF drug treatments. As a result, we considered HRF could be a reliable biomarker of patients with intractable ME after treatments with anti-VEGF agents.

HRF may appear in vivo inflammatory components of ME, and HRF can be presumed to activate microglia [17, 18]. Inflammatory mediators and the accumulation of inflammatory cells induced subretinal and intraretinal fluid accumulation in macular edema [18]. Moreover, HRF, as an indirect clinical sign of inflammation, was involved in retinal inflammatory response [8, 18]. In addition, HRF, also as an earlier manifestation of microvascular damage, may be important in the risk assessment of the progression of DME and vision loss [8]. HRFs have also been found to be a sign of vascular hyperpermeability [19]. In animal studies, a VEGF blockade has been shown to inhibit or reduce microglia activation [17]. Recent studies showed that, after anti-VEGF treatment, retinal vessel hyperpermeability improved and the number of HRF decreased in DME patients [16]. In this study, the number of HRF significantly decreased, which can be explained, to some extent, by the blockade of vascular hyperpermeability. Additionally, there may be other inflammatory mechanisms involved in this process.

Research shows that the origin of HRF might be from activated microglia related with the inflammatory environment in age-related macular degeneration. Anti-VEGF treatment is associated with a reduction of HRF in DME, and HRF has been considered a marker of active inflammatory status [20]. The detection of inflammatory factors in aqueous humor is a new approach to assess the status of ocular inflammation [21]. In this study, we detected inflammatory factors in the aqueous solution of patients with intractable ME and further studied their relationship with HRF, thereby providing more evidence on HRF's involvement in the inflammatory pathological process of ME. The study showed the total HRF before treatments and the change in numbers of HRF after treatments were both significantly positively correlated with IL-8 and VCAM-1.

IL-8 is a well-known proinflammatory cytokine that acts as a neutrophil chemoattractant and a T-cell activator. Researchers have reported that IL-8 directly stimulates VEGF expression and the autocrine activation of VEGF receptor-2 in vascular endothelial cells. Some research also found the aqueous humor levels of IL-8 increased significantly in patients with DME [22]. Many studies have shown that IL-8 levels in the aqueous humor of DME patients may be associated with inflammation-induced damage to the blood-retina barrier. VCAM-1, an endothelial cell adhesion molecule, is upregulated to the endothelium in response to inflammation and transmigration into tissues [23]. The levels of VCAM-1 in the aqueous humor increased with the severity of diabetic retinopathy, which suggests that VCAM-1 may play a role in the progression of diabetic retinopathy [24]. Therefore, these cytokines have the potential to be used as biomarkers to predict the progress of inflammation in ME. In this study, for patients with ME prior to treatments, the higher levels of IL-8 and VCAM-1, the more HRF were found in their OCT images and the more significant the reduction in HRF after the treatments. Besides that, the change in numbers of HRF at the 90-degree location in the OCT images was significantly positively correlated with IL-8 and VCAM-1. We think that the OCT image at the 90-degree location may better reflect the inflammatory reaction of patients and also have better clinical significance for the prognosis evaluation of macular edema associated with inflammation.

The previous further studies showed that IL-8 was secreted by microglia [25], and VCAM-1 could increase microglial reactivity [23]. The correlation between the numbers of HRF and the levels of IL-8 and VCAM-1 in ME in this study also supported our hypothesis that HRF represented activated microglia. We observed that, in the pathological process of ME, as the permeability of retinal vascular and subretinal fluid increased, the levels of IL-8 and VCAM-1 increased, thus promoting the activation of microglia. After anti-VEGF treatments, retinal vascular permeability was improved, inflammatory factor expression was decreased, and microglia activation was decreased. According to our results, the whole pathological process can be monitored by comparing the changes in HRF seen on OCT before and after anti-VEGF treatments. To sum up, HRF could be a new, noninvasive biomarker of functional and structural responses to anti-VEGF treatments or other drugs targeting the inflammatory microenvironment in intractable ME.

This study has still several limitations. Since aqueous humor accumulation is an invasive operation, we could not obtain the samples of aqueous humor from patients, which results in that we could not compare the change in inflammatory factors in the aqueous humor before and after treatments. However, according to our results, HRF, as an indirect indicator, can be used to evaluate changes in inflammatory factors postoperation. Although we obtained some significant results from this not too large sample, a further study can be conducted to test and verify our results based on large enough samples in the future.

## 5. Conclusion

In conclusion, we have observed that the numbers of HRF in intractable ME significantly decreased after anti-VEGF treatments, and there was a positive correlation between the levels of IL-8 and VCAM1, and the numbers of HRF shown by SD-OCT. The marked change in HRF and the relationship with the inflammatory factors suggest that HRF can act as a clinical marker for inflammatory reaction in patients with intractable ME. Moreover, they can provide as an effective noninvasive imaging indicator for evaluating the effect of anti-VEGF on inflammation-related diseases.

## Figures and Tables

**Table 1:** Basic information and inflammatory factors in aqueous humor measurement.

No.	Anti-VEGF agent(s)	Previous injections	VEGF	BFGF	IL-6	IL-10	IL-8	VCAM-1
1	Ranibizumab	2	670.6	0	0	0	0	0
2	Conbercept	4	7.4	0	4503.1	1.5	86.5	0
3	Conbercept	3	17.8	8.8	4.6	0.4	3.3	38.7
4	Conbercept	3	4.9	12.4	5.5	0.6	4.2	113.3
5	Ranibizumab	2	34.3	8.8	9.6	0	12.3	523.7
6	Conbercept	3	6.3	11.6	18.2	0	10.8	544.5
7	Conbercept	2	188.2	11.3	53.5	0	26.6	391.8
8	Ranibizumab	4	38.7	7.4	0	0	4.2	136.9
9	Ranibizumab	2	115.4	10.2	15.7	0	18.9	459
10	Conbercept	3	49	6.8	23.7	0	14.1	149.4
11	Conbercept	3	19.1	0	11.3	0	26.2	352.9
12	Conbercept	3	5.9	0	2.4	0	0.2	78.3
13	Ranibizumab	3	64.6	9.7	17.1	0	26.9	285.3
14	Ranibizumab	2	90.1	6.7	101.8	0	62.3	2867.1
15	Ranibizumab	3	101.2	6.5	4.5	0	18.3	197.2
16	Ranibizumab	3	140.4	0	91.7	0.6	35.1	843.1
17	Ranibizumab	3	308.3	13.7	22.4	1.1	25.5	704.5

The unit of inflammatory factors is pg/ml.

**Table 2:** Foveal thickness and HRFs in OCT images before and after anti-VEGF treatment.

No.	Before treatment					After treatment				
	HRF-90°	HRF-180°	HRF-45°	HRF-135°	Foveal thickness	HRF-90°	HRF-180°	HRF-45°	HRF-135°	Foveal thickness
1	3	2	4	2	177	2	1	1	4	165
2	6	7	7	9	619	0	1	2	2	162
3	4	0	0	4	187	0	1	1	1	167
4	0	0	0	1	351	0	0	2	0	162
5	5	7	7	6	540	2	0	1	1	179
6	1	1	3	1	643	1	0	0	1	566
7	3	3	8	2	221	1	0	2	0	197
8	8	3	6	3	521	0	1	2	0	235
9	2	1	2	3	307	0	2	3	2	214
10	3	1	3	2	684	0	0	0	0	381
11	8	9	5	4	716	4	5	7	2	651
12	1	0	2	0	566	1	0	0	0	469
13	0	15	4	8	322	1	7	1	3	259
14	20	7	14	2	209	8	5	6	1	184
15	1	3	0	2	272	0	0	2	1	199
16	3	0	0	4	195	0	0	0	0	162
17	1	0	2	0	201	0	0	0	0	185

The unit of foveal thickness is *μ*m.

## Data Availability

The data used to support the findings of this study are available from the corresponding author upon request.
